# Imaging optimization of FIB cross sections for reliable porosity analysis of PEMFC cathode catalyst layers

**DOI:** 10.1186/s42649-026-00142-w

**Published:** 2026-08-28

**Authors:** Ji Hee Kwon, Jinnyeong Jo, Byeong-Seon An

**Affiliations:** Analysis Center for Energy Research, Korea Institute of Energy Research, Daejeon, 34129 Korea

**Keywords:** PEMFC, Cathode catalyst layer, FIB-SEM, Porosity analysis, Cryo-FIB

## Abstract

Focused ion beam–scanning electron microscopy (FIB–SEM) was used to investigate the effects of acceleration voltage, beam current, and cryogenic conditions on the cross-sectional preparation of proton exchange membrane fuel cell (PEMFC) cathode catalyst layers. Milling at 30 kV and 0.26 nA effectively minimized curtaining artifacts and produced an apparent cross-sectional pore morphology similar to that obtained by cryogenic milling, indicating that optimized room-temperature milling can provide cross sections suitable for consistent two-dimensional (2D) pore-area analysis under the conditions examined.

Representative FIB–SEM cross-sectional images of PEMFC cathode catalyst layers prepared under different FIB milling conditions: (a–c) 5 kV at room temperature, (d–f) 30 kV at room temperature, and (g–i) 30 kV under cryogenic conditions (− 150 °C). The indicated voltage and current values represent the FIB acceleration voltage and ion beam current used for milling, respectively. The red dashed box in (f) highlights the optimized room-temperature milling condition (30 kV, 0.26 nA) used for comparison with the corresponding cryogenic condition in (i).

## Description

Focused ion beam–scanning electron microscopy (FIB–SEM) is widely used to characterize the pore structure of proton exchange membrane fuel cell (PEMFC) catalyst layers; however, milling-induced artifacts can substantially affect the interpretation of cross-sectional microstructures. Commercial Gore membrane electrode assembly (MEA) samples were used as representative PEMFC electrodes, and the cathode catalyst layers were selected for FIB cross-sectional preparation. The specimens were mounted on standard 3.2 mm pin-mount SEM stubs using conductive carbon tape. Cross-sectional preparation and microstructural analysis were performed using a Helios G5 UX focused ion beam system (Thermo Fisher Scientific, USA). For all milling conditions, a W protective layer was first deposited over the region of interest using the ion beam with a nominal deposition volume of 10 μm × 3 μm × 0.8 μm at 30 kV and 2.6 nA. Rough milling was then performed using a 15 μm × 15 μm × 9 μm milling pattern at 30 kV and 9.9 nA. Following rough milling, fine milling was performed using a 15 μm × 0.3 μm × 9 μm milling pattern under the conditions shown in the figure. At room temperature, fine milling was performed at 5 kV using beam currents of 11, 0.38, and 0.21 nA and at 30 kV using 9.9, 0.44, and 0.26 nA. For cryogenic preparation, the specimen stage was maintained at − 150 °C using a liquid-nitrogen cooling system, and fine milling was performed at 30 kV using beam currents of 9.9, 0.44, and 0.26 nA. For each condition, fine milling was repeated approximately five times to expose successive cross-sectional planes, with FIB–SEM images acquired from the resulting cross sections.

**Figure:**
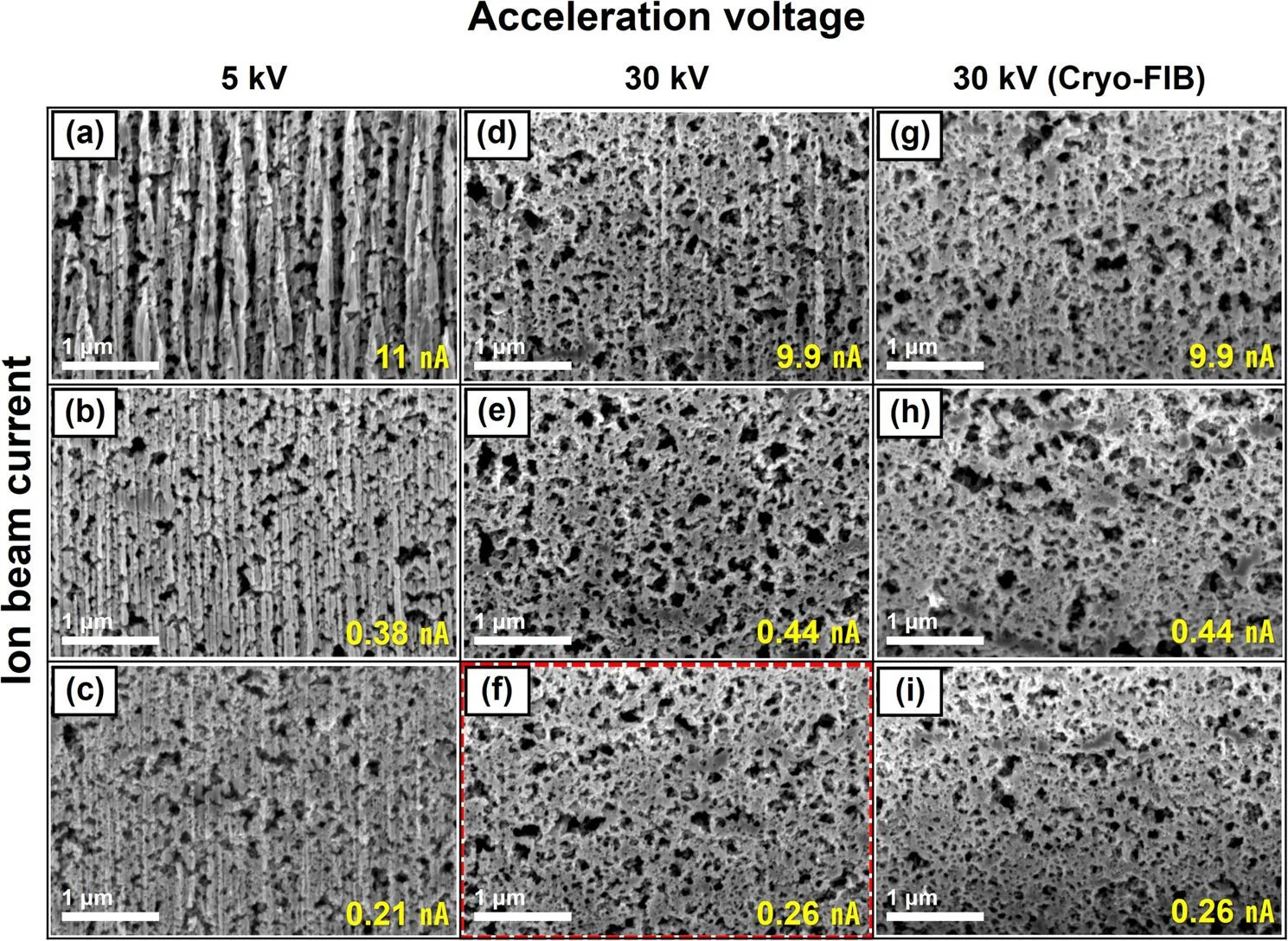
Representative FIB–SEM cross-sectional images of PEMFC cathode catalyst layers prepared under different FIB milling conditions: (**a**–**c**) 5 kV at room temperature, (**d**–**f**) 30 kV at room temperature, and (**g**–**i**) 30 kV under cryogenic conditions (−150 °C). The indicated voltage and current values represent the FIB acceleration voltage and ion beam current used for milling, respectively. The red dashed box in (**f**) highlights the optimized room-temperature milling condition (30 kV, 0.26 nA) used for comparison with the corresponding cryogenic condition in (**i**)

The most prominent feature of the image is the marked difference in cross-sectional quality observed at different acceleration voltages. Pronounced vertical curtaining artifacts are evident in the 5 kV images regardless of beam current, obscuring pore boundaries and reducing image contrast. In contrast, milling at 30 kV largely suppresses these artifacts, allowing the porous catalyst-layer microstructure to be visualized more clearly. Progressively reducing the beam current further improved surface smoothness and pore-edge definition, resulting in cleaner cross sections suitable for image-based 2D porosity analysis (Fager et al. 2020; Holzer et al. 2004; Liu et al. 2023).

The optimized room-temperature preparation (30 kV, 0.26 nA) produced an apparent cross-sectional pore morphology visually similar to that obtained by cryogenic milling at − 150 °C. For quantitative comparison, pore-area fractions were measured using ImageJ software (National Institutes of Health, USA). Three selected FIB–SEM cross-sectional images were analyzed for each of the optimized room-temperature and cryogenic milling conditions (30 kV, 0.26 nA), with one 2 μm × 2 μm ROI selected from each image. The selected ROIs were converted to binary images in ImageJ using the Default thresholding method with a fixed intensity threshold range of 0–65, which was applied consistently to all analyzed ROIs. The apparent 2D pore-area fraction was calculated as the segmented pore area divided by the total ROI area. The resulting values were 77.5 ± 1.6% and 79.9 ± 4.0% for the optimized room-temperature and cryogenic preparations, respectively, and are reported as the mean ± standard deviation of the three ROIs. The measured values should be interpreted as apparent 2D pore-area fractions derived from image segmentation and were used for relative comparison between the milling conditions rather than as measurements of bulk or three-dimensional catalyst-layer porosity. Under the conditions examined, these results indicate that optimized room-temperature milling can provide cross sections suitable for consistent 2D pore-area analysis. Rather than introducing a new milling strategy, this image illustrates how FIB milling conditions influence the observed cross-sectional morphology of porous catalyst layers and provides a practical visual reference for FIB–SEM imaging of PEMFC catalyst layers.

## Data Availability

The datasets generated and/or analyzed during the current study are available from the corresponding author on reasonable request.
